# Equine parasites: diagnosis and control - a current perspective

**DOI:** 10.1186/1756-3305-2-S2-I1

**Published:** 2009-09-25

**Authors:** Donato Traversa

**Affiliations:** 1 Faculty of Veterinary Medicine, University of Teramo, Italy

## Introduction

This Supplement to *Parasites & Vectors *titled "Equine parasites: diagnosis and control - a current perspective" is intended to provide a comprehensive collection of articles covering our current knowledge of cyathostomin and other roundworm infections in horses, and to be a single resource for information on diagnostics, prevalence, treatment and resistance of cyathostomins. The control of nematodes in horses has been challenging for some years and there is general recognition that improvements should be made to commonly used worm control programmes. These improvements should be based on scientific knowledge of the parasites, appropriate diagnostic techniques and should take into account the presence or potential selection for resistance to available anthelmintics for these parasitic groups.

Many of the articles in this Supplement come from a workshop on Equine Cyathostomins that was held at the Faculty of Veterinary Medicine of Teramo (Italy) on May 20^th^, 2009. The purpose of the workshop was to provide, for the first time in Italy, a hub of knowledge dedicated to these important horse parasites for undergraduate and postgraduate students and veterinary practitioners. The information presented in this meeting resulted from a large-scale study carried out in three European countries in 2008 on the diffusion of cyathostomins in horse properties and on the prevalence of anthelmintic resistance in their populations. This study has been supported by Fort Dodge Animal Health and scientifically coordinated by the team of Animal Parasitic Diseases at the Faculty of Veterinary Medicine of Teramo in Italy. In Germany the study was carried out and coordinated by the team of Animal Parasitic Diseases at the Faculty of Veterinary Medicine of Hannover, in UK, with assistance from the FDAH technical team and in Italy, with the participation of the parasitological teams at the Faculties of Veterinary Medicine of the Universities of Bari, Pisa, Padua and Udine.

The large amount of scientific data generated in these studies, and the importance of disseminating this information throughout the scientific community prompted us to organize such a meeting and subsequently prepare the present Supplement of *Parasites & Vectors*. The content of this Supplement expands beyond the European Project and, by inviting external contributors provides a global comprehensive review on cyathostomins and other equine nematodes with information including perspectives from the United States of America, where there is also concern on the control of horse parasites. The transport of horses across national boundaries for shows and performance competitions makes the issue of worm control and containment of resistant strains a matter of global importance. Indeed, the growing problem of anthelmintic resistance in horse cyathostomins has been reported in scientific journals and the presence of drug resistant populations represents a major hindrance for equine health and welfare worldwide. Resistance to Benzimidazoles (BZs) is globally spread, resistance to Tetrahydropirimidines (THPs) is increasing in prevalence in US and Europe as well.

Importantly, in the past few years, the first cases of reduced efficacy of macrocyclic lactones (MLs) have been reported.

The interest of horse parasitologists in control methods aiming both to control cyathostomin populations in the best way possible as well as preserving anthelmintic effectiveness as long as possible, has resulted in an increased awareness of the importance of understanding the biology and epidemiology of these parasites and the necessity for integrated prevention measures. Thus, this Supplement starts with a review paper on the biological and pathogenic significance of equine cyathostomins, followed by an article discussing how management practices may influence the presence and spread of these nematodes in horse yards and properties. In fact, adequate pasture hygiene, low stocking rates, age classes division and mixed grazing with other animals represent key measures to be integrated with the administration of parasiticides, along with the use of "selective treatments" rather than "interval dose program" (i.e. treat-all-animals) when appropriate.

The presence of anthelmintic resistance to the three major classes of anthelmintics found in Italy, Germany and UK is described and discussed in the article covering largest study carried out so far in the world on this problem. Data from 102 yards and 1704 horses were evaluated in three countries, i.e. 60 yards and 988 horses from Italy, 22 and 396 from the UK, 20 and 320 from Germany. The findings in Italy have been also discussed more in depth in a subsequent article, as particular regional differences in cyathostomin Faecal Egg Counts results and drug efficacies were found. Overall, the study showed that single and/or multiple drug resistance in equine cyathostomins is present in the three examined countries, with high level for BZs and/or THPs. MLs proved to be the most effective drugs, with some evidence of efficacy reduction for ivermectin and highest activity of moxidectin. However there was a single case of reduced efficacy to this product in Germany, highlighting need to use this compound wisely in order to preserve its use. Therefore, an article describing the chemistry, pharmacokinetics, efficacy and safety of moxidectin in horses is also presented, in order to guide rational future use of this molecule.

In past years, powerful insights into nucleic acids-based and *in vitro *diagnosis for horse small strongyles have been also gained, together with experimental evidence. It is generally recognized that neither current diagnostic methods nor treatment programmes are ideal. New detection and identification methods to measure resistance more accurately will help develop better and more reliable control measures. All authors highlight the need to move away from rote nematode control programmes and to the implementation of control measures based on the understanding of the biology and epidemiology of the parasites, the management practices used, and the particular characteristics of the anthelmintics available. The potentially valuable role of the veterinarian in developing such programmes is recognized.

We hope that the Teramo workshop and this Supplement are one of the next steps for improving health and welfare of affected horses in the decades to come. This improvement will only be possible with integrated and open discussion between the academia, industry and practicing veterinarians on current scientific knowledge as well as on future trends and needs concerning pathogenetic significance, epidemiology, diagnosis, and control of cyathostomins.

May this Supplement prove stimulating to future discussions and work on this topic.

## Competing interests

The author declares that they have no competing interests.

